# Glaucoma Quality of Life

**DOI:** 10.1155/2017/4257151

**Published:** 2017-07-18

**Authors:** Antonio Maria Fea, Fritz Hengerer, Carlo Lavia, Leon Au

**Affiliations:** ^1^Dipartimento di Scienze Chirurgiche-Clinica Oculistica, Università di Torino, Turin, Italy; ^2^Goethe University of Frankfurt, Frankfurt am Main, Germany; ^3^Manchester Royal Eye Hospital, Central Manchester Foundation Trust, Manchester, UK

Growing attention has been given to the quality of life in several fields of medicine. The concept of quality of life is not unknown to the glaucoma culture if we recall that the European Glaucoma Society Guidelines state “the goal of glaucoma treatment is to maintain the patient's visual function and related quality of life (QoL), at a sustainable cost.”

Nevertheless in the past, more attention has been given to quantitative matters as IOP, visual field, and optic nerve because those were the ones that can be directly modified and physicians were less interested in qualitative and subjective measures as the QoL. Measurements of QoL, on the other hand, tend to be time consuming and can be strongly influenced by other factors (general physical health, psychological state, personality, relationships, wealth, etc.), which are not necessarily related to the disease itself.

However, there is no doubt that when the physician pays attention to the claims of the patients, he will realize that most of their questions are related to everyday issues such as “will I be able to drive my car? Will I be able to be independent in my daily tasks within my home? And will I be able to go the supermarket two blocks from my house? Will I have problems reading?”

The same questions are relevant for insurances and governments because the disability caused by any disease will help determine the level at which the benefits of screening outweigh costs and decide which patient should be treated and how aggressive treatment should be. Furthermore, knowing the degree of disability can potentially help to increase patient safety with appropriate guidelines, to recognize patients who can benefit from rehabilitation and evaluate the efficacy of those measures.

Since the first studies on quality of life in glaucoma, several areas of interest have been identified and explored: (1) determining the symptoms that are more bothersome for the patients and correlating them with the stage of disease; (2) testing and building better methods to investigate the impact of glaucoma on the QoL; (3) assessing the impact of glaucoma on mental status; and (4) analyzing the impact of different therapies on the QoL of glaucoma patients.

1. Thanks to recent investigations, we are now aware that vision defects in glaucoma patients are not as simple as the traditional view of peripheral vision loss, but they affect several aspects related to a generally decreased image quality including glare, letters appearing faded when reading, and needing more light. Glaucoma patients gave the higher importance to tasks involving central and near vision (reading) and to mobility outside the home, whereas the most frequent complaints were difficulties related to lighting and in particular adapting to different levels of light. Common complaints also included difficulty in walking, stair climbing, face recognition, and driving. The relative importance of these problems is correlated to the degree of visual function and to the age of the patient. The correlation coefficients for the lower paracentral and lower peripheral VF of the better eye were the highest for several subscales, such as general vision, near vision, distance vision, social function, mental health, role limitation, and driving. Although the best correlations were generally found in the visual field of the better eye, the best metric to relate disease severity to disability is still a matter of debate and further work is needed in the field.
2. QoL assessment traditionally used patient-reported-outcome (PRO) questionnaires. More than 30 vision-specific PRO measures have been developed in the context of glaucoma and can be classified in three categories: PROs addressing functional status related to vision, PROs addressing overall QoL, and PROs assessing other factors related to disease and treatment (i.e., symptoms, side effects, adherence, satisfaction, and self-efficacy). Several studies failed to demonstrate a correlation between QoL and glaucoma severity especially when PROs were not specifically designed. Research on the most appropriate type of PRO has been very active. The information gathered with questionnaires are subjective and influenced by many factors other than the disease, including emotions, concentration, personality, and desire to please or to mislead. Furthermore, data from the SEE project showed that 10% of the subjects have differences between their perception on their ability to perform activities and their actual performance. Nevertheless, responses to questions about visual ability seem to correlate with clinical objective measures, which suggests that in the future, it may be possible to use them to define subgroups in the overall population. An active area of research in the last few years was the development and testing of standardized, performance-based measures of function performed in a clinical setting. Potential implications of the disagreement between subjective and objective testing will be to investigate why some patients have discrepancies between self-reported and performance-based tests. The knowledge of what an individual with a specific vision problem can actually do opens the way to develop tools to improve his performance, lessen his problems, and actively improve his quality of life. Another potential and poorly explored area is to follow prospectively the patients to understand how attitudes and QoL modify as the disease progresses, as the patient ages, or as other subjective or objective factors (wealth, social relationships, activities, etc.) change.
3. Glaucoma doctors always had the clinical impression that some psychological traits are typical of the glaucoma patients, but several papers demonstrated that glaucoma is a significant predictor of depression after adjustment for demographic factors and multiple comorbidities with a prevalence estimated around 10%. The finding that objective measures are not correlated to depression should alert clinicians that a high prevalence of depression may be present even among patients without clinically significant visual disabilities. Counseling regarding the generally slow progression rate of the disease may result in a decreased burden from depression.
4. The impact of new therapies, minimally invasive surgical procedures, and slow drug-releasing implants on the patient's QoL will certainly be a rapid growing field of investigation. The research in this field may potentially guide the researchers to select new therapies that have minimal effect on the quality of life and the agencies to evaluate the general and economic impacts of these new therapies on the glaucoma patients.

We decided to dedicate this special issue to QoL with the aim to shed light on the quality of life of glaucoma patients and attract attention of the scientific community to pursue further investigations leading to a rapid development of this field.

A better understanding of patient-reported QoL can improve the relationship between the patient and the physician and enhance adherence in choosing the treatment options on the basis of the patient profile.

